# “What Is the Matter With Me?” or a “Badge of Honor”: Nurses’ Constructions of Resilience During Covid-19

**DOI:** 10.1177/23333936221094862

**Published:** 2022-05-04

**Authors:** Anna Conolly, Ruth Abrams, Emma Rowland, Ruth Harris, Keith Couper, Daniel Kelly, Bridie Kent, Jill Maben

**Affiliations:** 1 University of Surrey, Guildford, United Kingdom; 2 King’s College London, London, United Kingdom; 3 University of Warwick and University Hospitals Birmingham NHS Foundation Trust, United Kingdom; 4 Cardiff University, Cardiff, United Kingdom; 5 University of Plymouth, Plymouth, United Kingdom

**Keywords:** nursing, resilience, qualitative research, distress, COVID-19, UK

## Abstract

It has long been known that nursing work is challenging and has the potential for negative impacts. During the COVID-19 pandemic most nurses’ working landscapes altered dramatically and many faced unprecedented challenges. Resilience is a contested term that has been used with increasing prevalence in healthcare with health professionals encouraging a “tool-box” of stress management techniques and resilience-building skills. Drawing on narrative interview data (*n* = 27) from the Impact of Covid on Nurses (ICON) qualitative study we examine how nurses conceptualized resilience during COVID-19 and the impacts this had on their mental wellbeing. We argue here that it is paramount that nurses are not blamed for experiencing workplace stress when perceived not to be resilient “enough,” particularly when expressing what may be deemed to be normal and appropriate reactions given the extreme circumstances and context of the COVID-19 pandemic.

## Background

Pre- COVID-19, research into nurses’ wellbeing has highlighted the intense pressure experienced by the nursing and midwifery workforce across the globe (Christodoulou-Fella et al., 2017). When compared to the rest of the UK workforce, nurses are at greater risk of work-related stress, burnout and mental health problems such as depression and anxiety (Kinman et al.’s, 2020). The experience of many nurses in the UK and internationally has been viewed as creating a toxic cocktail of unmanageable demands and limited autonomy (Christodoulou-Fella et al., 2017; Kinman et al., 2020; World Health Organization [WHO], 2006, 2021). The unprecedented working conditions experienced by nurses during the COVID-19 pandemic have further exacerbated this (Couper et al., 2022). The pressure widely felt among nurses to not disclose difficulties or emotions may be a main contributory factor as to why the notion of resilience has been keenly adopted in healthcare organizations (Kunzler et al., 2020).

## Emphasis on the Resilience of Individual Staff to Cope With Stress

With its Latin origins meaning “to spring back,” the term resilience can be defined as the ability to recover quickly from difficulties (Cooper et al., 2020). In the scientific community, resilience is frequently referred to in terms of how easily a material returns to its original shape after elastic deformation (Gorse et al., 2012). Psychological research has examined the concept of resilience with varying groups who have suffered psychological trauma, for example, children (Garmezy et al., 1984), patients with chronic illness (Guest et al., 2015) and trauma victims (Anderson et al., 2012). This research has tended to highlight the potential for promoting resilience and positive adaption to lead to improvements in mental health across many contexts (Cooper et al., 2020). Resilient individuals from the above studies were found to possess high levels of self-esteem, an easy temperament and a supportive environment. Much nursing resilience research has endeavored to assess the effectiveness of resilience building interventions (Delgado et al., 2017). In their recent review of the concept of resilience in nursing literature, Cooper et al. (2020) found that there was no universally agreed definition of resilience but the propensity to overcome adversity, to adapt and adjust, and to possess good mental health featured prominently in discussions of resilience. The cluster of attributes most frequently defined in the nursing literature as being associated with resilience are social support, self-efficacy, work-life balance/self-care, humor, optimism, and being realistic (Cooper et al., 2020).

## Resilience and Nursing

Nursing resilience studies have tended to define resilience as a personality trait (Delgado et al., 2017), with a focus on notions of adversity, (difficult or unpleasant situations) and positive adaptation (Cooper et al., 2020). However, studies that adopt such personalized, narrow, fixed notions of resilience can be critiqued. Within the field of behavioral science personality traits or attributes can be viewed as also having environmental causes (Biglan & Hayes, 2015). For example, Biglan and Hayes (2015) highlight how personality theory has been used to try to predict behaviors, but this has ignored how supposed personality traits, such as conscientious behavior, can be taught through organization training and the reinforcing (or rewarding) of conscientious behavior. Behavioral scientists argue that the identification of personality traits does not enable the identification of environmental causes of behavior nor how to alter the environment to remedy behavioral or workplace problems (Biglan & Hayes, 2015; Chiesa, 1992; Skinner, 1971). Similarly, little attempt has been made in many nursing studies of resilience to measure or define work-based adversity with few multiple site-investigations as to whether differences in resilience are associated with environmental causes such as different ways of working or challenging situations that are unreasonably demanding due to rising demand compounded by a lack of staff or resources (Morse et al., 2021; Traynor, 2017). One such situation is the impact of the COVID-19 pandemic on health systems worldwide.

## Increased Pressures Due to COVID-19

The COVID-19 pandemic exacerbated pre-existing pressures on the nursing workforce and research into the effect of the COVID-19 pandemic on healthcare staff has identified new stressors such as fears of contracting a highly infectious disease, concerns about staff shortages, insufficient personal protective equipment (PPE), navigating unfamiliar clinical settings or systems of care due to redeployment and lack of organizational support (Greenberg et al., 2021; Labrague & de Los Santos, 2020; Lapum et al., 2021; Maben et al., in press; Ohta et al., 2020; Ustun, 2021). Many of these pressures arising from COVID-19 can be viewed as organizational or systemic in nature. With the increase in stressors that nurses experienced during COVID-19, rates of burnout, PTSD and other mental ill-health have led to staff sickness and thus staff shortages and an exodus of nurses leaving the profession (Health and Social Care Select Committee, 2021; Maben et al., in press). Given the unprecedented challenges presented by COVID-19 there was therefore a need to better understand the nurse’s conceptualization of their wellbeing needs which was the aim of the qualitative study we undertook (see methods description below). Whilst analyzing our data the concept of resilience was constantly referred to by the nurses with whom we spoke. Therefore, this paper is specifically concerned with whether resilience was, and is, viewed as a helpful or detrimental concept during COVID-19, a time of acute pressure for nurses in the UK and internationally.

## Methods

### The Parent Study

The Impact of Covid on Nurses (ICON) parent study was developed by members of the UK Royal College of Nursing Research Society and aimed to explore the range of experiences of nurses working at the “frontline” of the COVID-19 pandemic and the possible impacts on their psychosocial and emotional wellbeing (Couper et al., 2022).

The sample was recruited through an opt-in method with a sub-sample of participants who had completed two ICON national nurse and midwife surveys (April and May 2020) in the parent study and who expressed an interest in being contacted to take part in a qualitative interview about their COVID-19 experiences (Couper et al., 2022).

### Qualitative Interview Study Sample

From those indicating a willingness to take part (*n* = 115) the participants were purposively sampled using quota sampling (Robinson, 2014) which enabled maximum variation within the sample of nursing roles, ages and experience, differing grades,^1^ specialties, settings and parts of the UK (*n* = 27 interviewees). This breadth of sampling was utilized to attempt to explore UK nurses’ COVID-19 experiences in a range of care settings and different regions, which may have had variations in prevalence of COVID-19, the impacts of redeployment (being moved from their “normal” role to work elsewhere) on those redeployed and the impacts of working alongside nurses who were.

Of the 18 nurses in our sample who had been redeployed during the pandemic, seven were redeployed to ICU (Intensive Care Units). Although the purposive sampling method was successful overall in procuring participants from a diverse range of geographies, level of experience, clinical specialties and job roles, the participants were mainly female (all participants except one were women) and only three were from ethnic minority groups.

### Data Collection

Qualitative in-depth narrative interviews (*n* = 27) (Maben et al., in press) were undertaken on two occasions. First interviews occurred in July 2020 (*n* = 27) and second interviews in December 2020 (*n* = 25). The aim of the qualitative arm of the study was broad, to explore the impacts on nurses of working during COVID-19. In both the generation and the analysis of our qualitative data we were concerned with the concept that “the whole is greater than the sum of its parts” or “Gestalt” (Hollway & Jefferson, 2013, p. 68). We thus took steps to counteract data fragmentation and to protect participants’ narrative arcs through specific analysis techniques (see below). Interviews were conducted by six experienced qualitative researchers (four of whom are professors of nursing and one also worked clinically during the pandemic). All of the interviewers, except one, were female. All interviews were conducted remotely via video conferencing platforms. Regular team meetings, from the study’s inception to the end of the analysis ensured consistency of epistemology, interview and analytic approach. An interview topic guide was used loosely, but as in narrative interviews, the researchers invited participants to “tell me what happened” and allowed them to speak without interruption (Greenhalgh et al., 2005). We followed respondents ordering and phrasing to allow participants to discuss areas they perceived to be relevant (Greenhalgh et al., 2005; Harding, 2006; Hollway & Jefferson, 2013).

### Ethics

Ethical approval was received from the University of Surrey ethical governance committee (FHMS 19-20 078 EGA CVD-19). The research team have considerable experience in conducting interviews on sensitive and distressing topics and offered interviewees opportunities to pause or stop if needed. A list of wellbeing resources and the opportunity to speak with a member of the research team after each interview were provided to participants to facilitate access to further support if needed. All interviews were anonymized and pseudonyms assigned.

### Data Analysis

Data analysis was initiated by becoming immersed in the data. Data were analyzed in two ways: (1) NVivo 12 was used to develop inductive codes and themes across each of the two datasets (first and second interviews); and (2) participant interview summaries, or pen portraits, were written to avoid fragmentation of the data, which can occur with systematic coding (Hollway & Jefferson, 2013). This helped preserve the narrative for each participant and which supported longitudinal analysis. In terms of rigor, inter-rater reliability (Elliot, 2018) in the form of corroboration and legitimatisation of coding, was achieved by AC leading the coding process with a sub-sample of transcripts selected for additional analysis by ER (Charmaz, 2006; Mills et al., 2006). A selection of the pen portraits were also “moderated” by a second researcher, and these, along with the secondary level themes, became essential tools to aid a holistic analysis of each participant (Hollway & Jefferson, 2013). We utilized narrative analysis, a case study approach, which identifies segments of text that take the form of narrative (Riessman, 1993; 2002). Narrative analysis enables the examination of structural and linguistic features to analyze how they support particular interpretations of lived experience (Riessman, 1993; 2002). The approach preserves the integrity of the narrative and expands the basis for interpretation: how experiences are talked about is as important to interpretation as what is said (Edvardson et al., 2003).

Whilst immersed in the nurses’ narratives, we were struck by the way that many of the nurses talked about their resilience, as they struggled with extremely adverse working conditions. This article presents three case studies from three separate ICON qualitative participants. These case studies were chosen by the research team because they serve as exemplars of the full data set, illuminating two diametrically opposing resilience narratives, one in which resilience is perceived as a badge of honor and the other in which resilience is viewed as a stick to beat oneself with. Whilst opposing, both narratives explore a relationship between passivity and resilience. In the third case study we explore the relationship between resilience and agency. Presenting the data through three case studies allows us to fully interrogate the biographical narratives of three of our participants and explore how resilience is enacted in practice.

### Case Studies of Resilience

For the purposes of this article, we have presented examples of our data on resilience in three case studies, to enable “critical comparisons of both the form and content of narrative data that alternative approaches do not capture” (Thomson et al., 2002, p. 352). Through this method, biographical and life experiences can be taken into account to explore, test and refine theoretical ideas about the relations between core concepts (Hollway & Jefferson, 2013; Kvale, 1999; Mitchell, 2000). The case study approach employed here enabled us to analyze and illuminate how the nurses in our study conceptualized resilience, how it was embedded into their identity and the consequences and effects of these constructions. The three exemplar case studies discussed below are individual nurses, and we use quotes from their narratives. After analysis of all our data, and inter-rater reliability on the codes, themes and pen portraits, these three case studies were chosen, because they can be conceptualized as a set of categories defining types of a social phenomenon (Douglas, 1967; Flyvbjerg, 2006). We discovered that, on the whole, nurses in our study spoke of resilience in one of two ways, both of which were somewhat passive, either viewing themselves as very resilient, of which they were proud, or viewing themselves as normally resilient but were coping less well and questioning why their resilience had slipped during the pandemic. For those experiencing the latter, this feeling of not coping frequently left our participants feeling like they were unable to take action to find support. The third case study represents a contrary or negative case which we use to highlight the relationship between resilience and agency. Contrary cases can be characterized as consciously seeking and explaining outliers (negative cases) in the data, exemplifying an unusual or alternative stance which stands them apart from the rest (Guba & Lincoln, 1994). Our contrary case is non-typical due to the participant’s use of agency. These three case studies are presented to illuminate what it is to be resilient as a nurse in practice during unprecedented times.

## Findings

During our data analysis we discovered that resilience was often referred to by nurses as existing on a spectrum; at one end, those that were able to bounce back and better control their negative and destructive emotions had lots of resilience and at the other end there were those that were unable to keep going and/or control negative and destructive emotions at the other. On the whole nurses were keen to present themselves as having lots of resilience (exemplified by Sandra in case study one) and were puzzled and self-blaming when they were not resilient (such as Isabella in case study two).

## Three Case Studies of Resilience

### Case Study One: Resilience as a Badge of Honor: Sandra

Sandra was redeployed to ICU during the first wave of the pandemic and her COVID-19 experiences reflected a commonly held view across our data set that resilience equated to stoicism. For the majority of the nurses, exemplified here by Sandra, their “resilience” was intertwined with their professional identity:

I’m a very resilient person, I don’t really. . . there’s the odd patient that comes in that really moves you, like when kids come in to say goodbye, you do get quite emotional, but otherwise I don’t really struggle at work.

Similar to Kirk et al.’s (2021) participants who emphasized the importance of stoicism in their discussion of “emotion rules,” Sandra’s conceptualization of herself as a “resilient person” entailed “not struggling at work” which for her meant not letting the majority of her patients get to her emotionally. Previous research (e.g., Jackson et al., 2021) has highlighted the complex and multi-faceted nature of nursing work which can be understood as a composite of physical, emotional, cognitive, and organizational labor and the fact that much of this work is invisible (Allen et al., 2014). Sandra qualified her definition of resilience through reassuring the interviewer that she displayed emotion at extreme moments, such as when children say a final goodbye to their parents, but she made it clear that emotion does not really tend to overcome her and therefore her approach to nursing embodies her notion of resilience.

Sandra had spent over twenty years working on ICU before spending the year prior to COVID-19 as a critical care outreach nurse. She was redeployed full time back to the same ICU she had previously worked in, so was familiar with the workspace to which she was redeployed. However, due to the pandemic, the familiar working environment was no longer familiar, due to imposed restrictions on her freedoms as a result of infection control measures. These changes were disconcerting leaving her feeling both emotionally and physically depleted. Sandra powerfully described her time on ICU during the first wave of the pandemic:

Because of the shortage of PPE, we were only let out twice in 12 and a half hours. And then you had to decide whether you wanted to drink or eat or wee, or if you drank too much then you might need a wee and you couldn’t get out for a wee, and that was absolutely exhausting. I know we’re used to looking after ICU patients, but you throw in dehydration and overheating, it was quite unpleasant actually. I can remember having to move quite slowly around the bed space to stop myself getting too hot, because if you started getting too hot you started feeling quite claustrophobic with the mask and then visors and stuff on.

It is striking that, although she had considerable ICU experience, Sandra described her work during the pandemic using terms such as “unpleasant,” “absolutely exhausting,” and “claustrophobic.” Cooper et al. (2020) have referred to the notion of “antecedents,” or incidents which occur prior to the behavior, in resilience as representative of an adverse event which would have to be overcome. Certainly, for Sandra, similar to the majority of nurses we spoke to, the pandemic represented a very altered working landscape and can be viewed as an antecedent event (Chiesa, 1992). Many of the study participants referred to PPE shortages during the first wave of COVID-19 which meant that supplies were rationed. The systemic failing of PPE shortage contributed to the extremely difficult working environment Sandra, and others had to deal with, but Sandra herself did not overtly draw the link between the challenges to her emotional and physical wellbeing she was experiencing at work and her very tough working environment.

Physically the nursing work Sandra performed was very difficult, which she was aware of and she took certain steps to control, such as moving “slowly around the bed” to avoid overheating. She also referred to having to limit the amount she drank due to not having adequate toilet access due to limited PPE. Therefore, she referred to making conscious decisions, adjustments to her own behaviors, to mitigate the impact of her very tough working conditions and lack of vital protective equipment on her physical wellbeing. In doing so Sandra takes responsibility for her own working conditions and compensates for the failure of the organization to provide sufficient equipment. It can be argued that by making these adjustments Sandra was internalizing and attempting to mitigate the effects of working through the pandemic. At the end of her first interview Sandra referred to the physical impact the pandemic had taken on her:

I do feel breathless, and I don’t know whether that’s related to wearing a mask for a month or whether it’s anxiety.

As well as physical symptoms Sandra also referred to the emotional effects she was experiencing from working during the pandemic stating that she felt “quite depleted” and not “quite myself” and was less resilient than normal, being more emotional:

I have noticed that if I see something sort of slightly tender or kind, I’m quite quick to well up and you sort of feel overwhelmed by emotion.

In her second interview Sandra referred to attending a compulsory team day that had a wellbeing element, noting the need to share experiences within professional sessions and networks:

The clinical psychologist came, two of them came, and they had planned sessions and stuff. But actually, what they ended up doing was a debrief, almost (. . .) I think she just asked, you know, how was it for you? And then three hours later people were still talking, and that was quite interesting (. . .) But actually, people just get on and do things, and you don’t always empathise that everyone is struggling, you know, with different issues and things (. . .) but it was really eye-opening to listen to all of us speaking and how valuable that was to hear what people had been through.

The need felt by the nurses to share their experiences in the group setting described by Sandra reveals the possibility that support is needed and that perhaps resilience is waning for the nurses due to the difficult workscapes they have endured with COVID-19. The value to Sandra of attending the psychologist session resonates with other research, which has indicated that emotional expression or catharsis, particularly in groups, whereby individuals can reflect upon their emotional reactions, facilitates emotional adjustment, enhances mental health and life satisfaction, and can promote positive outcomes, thus, reducing the risk of emotional exhaustion for nurses (Kinman & Leggetter, 2016). It can be argued that acknowledging emotional vulnerabilities in a group situation such as the one described by Sandra, gives nurses the opportunity to step outside the normal emotion rules which dictate that nurses should be able to keep going come what may or show resilience (Kirk et al., 2021). Sandra, similar to the majority of the nurses we spoke to, revealed that she felt unable to share her COVID-19 working experiences with friends or family. Having good social and professional networks has been identified as one of the attributes most frequently associated with the concept of resilience in nursing literature (Foster et al., 2019; Taylor, 2019; Traynor, 2017). The absence of home and friend-based support during the pandemic potentially increased the importance of work-based support and support groups. However, the willingness of nurses to engage with debriefing or therapeutic processes can be limited and Sandra stated that the psychologist was having problems in getting nurses to attend her sessions. Whilst this might be due to challenges of finding time to attend, Sandra highlighted her perception of the tendency for nurses to be stoic and carry on, this being something of a “badge of honor” with links to their sense of self as being hardworking:

She [the psychologist] says she can’t get staff engagement. She’ll set up things and no one will come. Because nurses like to be resilient and you’re either coping or you’re not coping. And people wait until they’ve fallen off their cliff edge to get help. But actually, you can prevent it (. . .) you can be coping but still need support (. . .) and still feel better after you’ve done something and avoid getting to the point of where you’re not coping (. . .) but I think it’s worse for nurses, because nurses like to be martyrs as well, you know? Look how much work I’m struggling through, you know? (. . .) I haven’t had lunch until 4 o’clock, you know? It’s a bit of a badge of hardworking resilient-ness, maybe.

For Sandra, nurse resilience encapsulates not showing emotion, working “hard,” being a “martyr” and/or “struggling through.” This resonates with other studies which have identified the covert rules nurses operate under which include “no shirking” as “good” nurses undertake their fair share of hard physical work (Kirk et al., 2021). While Sandra outlines nurses’ desire to keep going, she also accentuates nurses’ capacity for complaint. Complaining, or the “badge of hard-working resilient-ness” enables nurses to express how hard they are finding their working conditions perhaps cementing their lack of agency. It is as if by complaining in an informal manner to each other the nurses feel validated or for Sandra “martyred,” and therefore no impetus or push exists for the nurses to implement their own agency to take issues further, for example with management. Previous literature has highlighted the pleasure found by nurses in repeating accounts of their apparently powerless position (Traynor, 2018). As Zizek (2005) notes, being a righteous victim can provide a sense of having a moral high ground. Badges are visible representations, worn to be displayed with pride for others to see. Sandra conceptualized nurses as wanting to endure difficulties at work until they are at a point of desperation or they have “fallen off their cliff edge.” The latter implies that resilience is dynamic and contingent, not a static state and that there is a point where resilience is eroded or is sufficiently depleted to result in poor mental health consequences.

### Case Study Two: “What’s the Matter With Me?” Resilience as a Stick to Beat Oneself With: Isabella

Isabella’s case study provides an example of how the concept of resilience can make those experiencing stress and similar psychological problems feel worse. The problems identified with the term resilience and its use in the NHS are summarized by Isabella below, as she questions, her own perceived individual failings. Isabella had considerable ICU experience, but had been “out of it for years,” but was re-deployed to ICU in the pandemic. Pre-COVID-19 she had believed she possessed more, or certainly an adequate supply of resilience:

And I feel like I’m pretty resilient, I’m pretty, I mean, I’ve worked ICU a long time, I’ve worked major trauma so I’ve seen some really nasty stuff and, you know, but this, I was a bit shocked at how it affected me, I just thought what’s the matter with me?

Here the negative aspects of resilience are clearly displayed as Isabella’s expressed self-blame reveals how feeling distress and not being resilient enough can be conceptualized as a personal failing, letting organizations and systems “off the hook” (Traynor, 2018). Isabella listed her previous experiences which she believed would have made her more able to cope with the stresses of the COVID-19 pandemic and be more resilient, including having worked in “ICU for a long time,” “major traumas” and “really nasty stuff.” In her narrative Isabella highlighted the prevalence of many systemic failings which her distress, or as conceptualized by Isabella, a lack of resilience, can be viewed to stem from. She also identified her distinct lack of power and agency. For example:

[It was] like being in the army, it’s like you didn’t know when you’re dispatched, you know where you’re going to be dispatched, who you’re going to be working with, when you could eat, when you could drink. And I remember I would come out, one awful shift, I’ll go through with you in a minute, but remember just thinking I’m going to wet myself, I have not had a drink since 7.30 this morning, I’ve not eaten since 6 o’clock (. . .) you just couldn’t and that I found really, really difficult and really challenging.

The working conditions resulting in a lack of agency that Isabella described above severely impacted on her ability to achieve self-efficacy, self-care and therefore her optimism and resiliency. These attributes have been labeled as essential criteria for resilience in nurses according to Cooper et al. (2020). Like Sandra, Isabella was clear that the reason she was given so few breaks was due to PPE rationing, which can be considered a system failure. She referred to the “insecurity” of not knowing when the next box of PPE was going to arrive as “really stressful.” Isabella described feeling powerless when she believed her PPE mask did not fit her correctly (she was alerted to this because her glasses were “steaming up”). She felt she could not raise concerns regarding inappropriately fitting masks because that would have taken her away from her shift, leaving her colleagues short staffed. The willingness to do one’s duty in spite of the personal cost is a feature that came through consistently in the narratives of those we spoke to and has previously been argued is essential for the continued normal functioning of the NHS (Traynor, 2017) and one which healthcare systems relied upon even more during the COVID-19 pandemic.

Unlike Sandra, who worked in the same place every day, Isabella’s sense of distress, and powerlessness, were compounded by not knowing on a daily basis which (of the several) ICUs she was going to be working in or who she was going to be working with due to a lack of continuity in her redeployment. Despite asking to be based on one ICU, she was regularly moved around, which did not allow her to become familiar with the team, the environment, or patients. Similar to many of those we spoke to who were redeployed, every day was a new stressful day with nothing familiar. Thus, similar to the majority of our other participants, there was “nothing grounding” about her COVID-19 working practices which she found really “challenging.” It seems unlikely that a conscious decision was taken at a managerial /system level to keep moving those re-deployed to different locations, however, when Isabella did query this asking to be kept in one location, her requests seemed to fall upon “deaf ears” (Jones & Kelly, 2014). It would appear that no safeguards were in place to keep re-deployed nurses in as few locations as possible, maximizing continuity for the safety of patients and staff. Her constantly changing working environment meant that Isabella frequently did not know where essential equipment was kept which was very personally stressful and had patient safety implications. She related one incident where she had to take a critical patient to radiography which felt “like a comedy of errors” due to the number of things that went wrong because of the chaotic nature of that “particular unit.” Isabella referred to what occurred as “really, really unsafe” and she felt “unsupported” and “alone” because “if anything happens to this patient, it would be down to me and I felt really, really vulnerable.” Therefore, Isabella’s distress, can be viewed to be a result of systemic/managerial failings during COVID-19 with potentially serious implications for patient care.

Isabella identified many other examples of managerial shortcomings, feeling unsupported and a lack of social support at work and the effect these had on her mental health. High levels of social support are frequently cited as being associated with high levels of resilience (Mealer et al., 2017). Isabella stated that “senior staff were sitting out” and permanent senior charge nurses (Isabella’s own grade), and above, who normally worked on the units where she was redeployed, were avoiding patient contact. Isabella stated “all of us [those redeployed] felt a bit disappointed by that, that actually we were almost like we were the cannon fodder.” Isabella also frequently referred to treats from the public being withheld from her and her redeployed colleagues by managers, which added to her feeling unsupported. She reported that the boost boxes (boxes that the public had donated to support nurses during the pandemic) were not shared equally among the staff instead being “taken away” into the office for the permanent staff, which to Isabella seemed “petty” and divisive. Isabella also referred to occasions where she was shouted at which added to her feelings of being unsupported and isolated and which perhaps contributed to her feeling less resilient:

I remember once I had a ventilated patient and someone in an empty bed space shouted at me to answer the phone and I thought, who speaks to anybody like that? I thought, I speak to nobody like that, (. . .) so I said, well, can you watch my ventilated patient while I go and answer the phone? I just thought, whoa.

The poor working conditions Isabella experienced during her COVID-19 redeployment, as a result of the environmental or managerial failings, were cited by her as the causes of her distress, which she linked to a lack of resilience. Thus, she has internalized external stressors as a personal failing. Traynor (2018) highlights two “entirely different sources of adversity” for nurses which can be viewed as antecedents, or the events that occur prior to the occurrence of the concept, (Walker & Avant, 2011) of resilience. These antecedent sources of adversity can be viewed as leading to the consequences of either experiencing negative psychological outcomes (if individuals do not have enough resilience) or the prevention of negative psychological outcomes (if individuals have an adequate amount of resilience) (Cooper et al., 2020). The first source of adversity involves dealing with the suffering of patients and their families, with the effects of extreme illness or mental distress, which can be viewed as intrinsic to nursing work itself (Traynor, 2018). It would seem that the “really nasty stuff” that Isabella referred to in her first extract—her pre-COVID-19 experiences—can be viewed as falling into this, intrinsic to nursing work, category. The second source of adversity identified by Traynor (2018), when writing about resilience, is a consequence of:

Political decisions, under-resourcing, poor management, dysfunctional and insecure organizations, disempowered nurse managers, sexism, racism in the workplace, which all result in understaffing, perhaps, and high turnover (Traynor, 2018: 7).

From her detailed descriptions of her working environment during COVID-19, Isabella’s experiences fall into this, the systemic failing category of nursing. Traynor (2018) has argued that structural adversity and challenging context intensifies the effect of the first type of adversity as nurses become less able to properly address the needs of the increasing number of patients they are required to care for. For Traynor (2018) nurses need to be able to cope with the first source of adversity to do their jobs, however the second source of adversity can be viewed as being beyond their control. Our data, along with other research, indicate that both sources of adversity existed for nurses during the UK during the COVID-19 pandemic (British Academy, 2021), significantly increasing the risks of nurses experiencing negative psychological outcomes and feel that they are not resilient enough. After her redeployment Isabella was left with an overwhelming sense of distress:

I did feel this is the worst time in my nursing career and I said that to people, and I just, I don’t know why, maybe the more I’m sort of talking about it maybe it makes sense, but I couldn’t work out why that was but it just felt like the worst time in my 30 years, worst thing I’ve ever done.

It is striking that Isabella classified her redeployment during the COVID-19 pandemic as the “worse thing” she has “ever done,” but equally striking that she “couldn’t work out why.” To the research team, analyzing her full narratives, the multiple system failings Isabella encountered, such as the shortages of PPE and how these were managed, along with a toxic team culture stand out as antecedents or triggers for the decline in her mental wellbeing, her feelings of self-blame and her asking what is wrong with me? All contributed to her feeling as she did. Isabella did speak to a counselor, but again, similar to the conceptualization of resilience as a personal trait, Isabella’s counseling experience was framed in terms of individual, not social or systems wide, experience. Isabella did however find the counselors words useful:

It was just about recognising when my stress bucket is full. I thought (. . .) that’s a good analogy. About nourishing my mental wellbeing, being kind to myself and using my strategies so like yoga, fitness, rest, my friends.

Isabella had reached her limit, she had experienced enough adverse antecedent events, for negative psychological outcomes to occur which left her feeling not resilient enough. The suggestion to “nourish” one’s “mental wellbeing” resonates with literature around neo-liberal self-help discourses where individuals are encouraged to “work on themselves – often at the exclusion of the social” (Riley et al., 2019, p. 5). Similar to the concept of resilience, the notion of self-help advocates that answers to problems can be found within, and therefore looks to remedy the individual’ failings, as opposed to systemic failings. The process of self-actualization is never complete with individuals expected to constantly work toward self-improvement with daily wellbeing tasks, striving for higher levels of health and self-realization, so that “self-actualization through transformative self-help remains an elusive goal” (Riley et al., 2019, p. 6). Nurses may never truly be “resilient enough” to cope with the systems failings that surround them in the toxic culture of the NHS, particularly in the maelstrom of a pandemic, with the consequence that they feel they are always to blame for a “lack of resilience,” which will inevitably lead to an increase in prevalence of negative psychological outcomes.

### Case Study Three: Systemic Failings But Pride in One’s Own Resilience: Amie

Amie was selected as our last case study example because she represents a contrary case. Whereas other participants, similar to Sandra and Isabella, “got on” with their situations, Amie was able to use her personal sense of agency to channel distress into action, and to challenge the situation in which she found herself. Having worked with older people for most of her career, Amie was the nurse manager of a care home during the pandemic. As a manager, Amie took pride in her own resilience, even though she encountered work-based stress due to system failings, but she also used the concept of resilience to question the abilities of her employees.

COVID-19 presented difficult systemic problems for Amie which led her to describe feelings of extreme distress, due to the systemic failings, yet pride in her own resilience in the face of such adversity. She confided that she had found the first wave of the COVID-19 pandemic very difficult due to PPE shortages. In her first interview, Amie referred to feeling like she had been “tricked” into accepting patients from hospital into her care home during the first wave of the pandemic. Unknown to Amie, most of the patients she was asked to take already had care home spaces prior to their admittance to hospital. Amie later found out that because these patients’ previous care homes were classified as COVID-19 free they no longer accepted patients who had been admitted to hospital. When Amie discovered the reason why their previous care homes had not accepted these patients, she described feeling as if she and her team had been:

Totally used. Here we were taking people in, believing that we were doing the right thing for the individual, they weren’t being left in hospital, believing that we were doing the right thing for the system, believing that we were doing the right thing for staff and existing family members living with us, because actually we were trying to keep the whole organisation financially viable. So, all of those things came from a point of view of trying to do the right thing for everybody. But then there was this sense of feeling that we were being used and abused by the system (. . .) And I just couldn’t believe that that was the situation. I couldn’t believe that people would act in that way.

The powerlessness, frustration and sense of being hoodwinked by her external managers that Amie described caused her considerable distress. However, Amie was able to use her feelings of distress to try to effect change. As a commissioned provider of care, Amie felt she was bullied by external colleagues for speaking out. She commented that her external managers:

They weren’t open and saying that this was the situation, they were doing it in a very, well, bullying (way), that’s what it was, it was bullying.

Amie described attending a zoom meeting where she turned her camera off because she cried throughout. Thus, the frustration and distress she experienced due to the systemic challenges she encountered affected her mental health profoundly. However, even though Amie was clear that her distress was being created by the systemic problems and her managers handling of the situation surrounding COVID-19, like Isabella, Amie attributed responsibility to herself, personally, for her management of her distress. However, unlike any other participants we interviewed, Amie spoke out and used her agency to highlight the issues with accessing PPE, whilst presumably knowing that this would anger her managers. Sandra and Isabella, like the majority of our other participants, coped with the situation but did not feel able to challenge it in such a direct manner, and certainly not by raising issues within their employers or to external bodies. Therefore, the different approaches to resilience are illuminated as Amie challenged the systemic causes of her distress whereas Sandra and Isabella in effect accepted them and attempted to get on with their situations. It is possible that Amie felt protected due to authority as a manager and her high levels of resilience, even though she must have known repercussions would be inevitable. In the extract below we can see the credit she gave herself for being able to bounce back (be resilient) from her “distraught” state and the need for some agency in difficult situations. She also questioned the resilience levels of staff members who were not able to do the same, suggesting resilience was waning in staff, yet she also worried about how to support her team:

I suppose I have been concerned for a long time that people don’t seem to have the resilience that they once had. At the moment we’ve got a lot of people that are talking about their anxieties. One of my staff who does suffer from [psychiatric illness] said to me, “Oh we should be able to have these feelings, we should be able to acknowledge our feelings.” I said, “Yeah, acknowledge them by all means, but we’re not going to dwell on them and we’re not going to be, “Oh it’s been awful, and then we can’t do anything.”“ (. . .) It’s been awful, but we now need to move on. I said, “Or is that your point, that you can’t move on, and therefore we’re throwing in the towel because it’s been so awful? Is that what you’re saying?” (. . .) [Name] said to me, because (. . .) we’d spoken I was distraught. And then when she spoke to me, (. . .) she said, “I cannot get over your resilience. Where do you get your resilience from?” I need to be mindful that that’s how I am, but everybody is not like that. So how do I support my team to move forward? Because we are all at different points, we all have different views, and how do we take that forward?

Amie’s frustration in her staffs’ wish to talk about their anxieties (encouraged as a good response by most mental health professionals) led her to question their levels of resilience. Amie’s response may be viewed as a coping mechanism as the required effort or extra emotional burden involved in supporting those “failing to cope” during the pandemic may have been too much for individual colleagues and teams to take on leading them to encounter more emotional distress. It is clear that Amie believed that her staff should just get on with it, even though, at the time of her distress, she did not just get on with her job, but instead took time to use her own agency to try to effect change. Amie elaborated in further interviews that she did offer her employees mental health support which she expected them to utilize. She stated her mantra was that “you can’t change something unless you do something about it,” and she urged her employees to take action, rather than using “depressive illness or anxiety as an excuse” for inaction. Amie’s agentic action to effect change was an unusual stance taken amongst our participants, it is clear from the above extract that, similar to Sandra and Isabella (and to our other participants), Amie had “bought in” to the NHS resilience narrative and expressed dismay at the notion of her staff dwelling upon their pandemic experiences. Amie wore the compliment that she received about her resilience similar to the “badge of honor” (mentioned by Sandra). Amie took pride in her resilience, after being so visibly distressed due to circumstances beyond her control, she appeared pleased for being commended on her own resilient nature and her ability to bounce back.

## Discussion

This paper contributes to current academic and policy literature by raising awareness of nurses’ stressors and psychological experiences during the COVID-19 pandemic, when, nurses felt alone, unsupported and even bullied. Our data shows that the concept of resilience has been internalized by many nurses within the UK health service to their detriment, preventing help-seeking for their mental health and for the adverse working conditions they experienced. Therefore, our unique contribution is a deconstruction and critique of a prevalent individualized resilience narrative identifying it’s considerable implications for nurses during a time of crisis, when the workforce has needed significant psychological support.

In the introduction we discussed the conceptualization of resilience as spring-like, or something that returns to original shape. This imagery suggests the stressor is temporary and short-lived. This was not the case for our participants, the stressors continued over a sustained period of time. It is therefore pertinent to query whether the term resilience is appropriate to use for nurses’ adaption to the current challenging working situation within the NHS, specifically during the COVID-19 pandemic. Although nurses working conditions pre-pandemic featured significant adversity due to issues such as exposure to shift work, interprofessional conflict, and death and dying (Happell et al., 2013; Hsieh et al., 2016; Mealer et al., 2017) the altered landscape of COVID-19 nursing work can be viewed as an antecedent event which would make nurses even more susceptible to stress and distress, decreased job satisfaction, decreased quality of patient care, decreased coping and reduced staff retention (Cooper et al., 2020). It is clear that during the COVID-19 pandemic the majority of the nurses we spoke to were not able to access, or had their access restricted to, many of the defining or protective attributes associated with resilience in the nursing literature such as self-efficacy, optimism, humor, work-life balance/self-care, being realistic, although they were able to access support networks (Cooper et al., 2020). As we have discussed in this paper, nurses also experienced an array of further, pandemic specific barriers, such as not having enough PPE, lack of breaks, the significant challenges of wearing PPE, managers working from home, relatives not being allowed to visit patients as well as mental and emotional overload. If we take into consideration Maslow’s (1943) seminal work around hierarchy of needs, where physiological needs (shelter, food, water, clothing and being safe) need to be met before self-actualization can occur, it is useful to posit the question: how could nurses possibly be resilient whilst working during COVID-19 when their basic physiological and safety needs were compromised?

The analysis and presentation of data through exemplar case studies in this article has enabled us to explore in depth, how, in the midst of a pandemic, some nurses perceived resilience as either a badge of honor, or a stick to beat themselves up with. Those who were redeployed seem to have identified themselves as having less resilience than those who were not redeployed, such as Amie, which emphasizes the importance of known colleagues and supportive teams for nurses. We argue that our data have revealed how, during “unprecedented times,” the concept of resilience, which has been utilized by health organizations and policy makers, has been internalized by nurses as a method of legitimizing emotional labor and organizational feeling rules (Kirk et al., 2021; Maben et al., 2006). These emphasize stoicism and, therefore for the majority, limited their agency, preventing them from speaking out or seeking change.

The overall unifying factor in the nurses’ talk of resilience in our study was their conceptualization of resilience as an individual responsibility, in keeping with the literature suggesting it is a personality trait (Happell et al., 2013; Hsieh et al., 2016; Mealer et al., 2017). Resilience interventions and courses can comprise practice-focused interventions (e.g., daily mindfulness sessions) and/or educational interventions (workshops and mentoring programs) (Delgado et al., 2017; Foureur et al., 2013; McDonald et al., 2013). Nursing-based resilience intervention research mostly rely on individual-based, not systems based, interventions and therefore on the actions individual nurses can take to develop and sustain resilience (Cooper et al., 2020; Foster et al., 2019; Taylor, 2019; Traynor, 2017; Virkstis et al., 2018). The majority of resilience-based initiatives within the UK NHS operate within this individual-based framework, as opposed to environmental foci. Therefore, it can be argued that much resilience-based nursing research is “psychocentric,” a concept which has been used to explain the individualization of human problems, through the enabling of victim-blaming in a reductionist, determinist, positivist and pathological individualist manner (Rimke, 2016). This approach effectively restricts narratives around mental health and wellbeing and entails overlooking wider social, political and cultural contexts. For example, a recent critical analysis of UK suicide policy found that the definitions of suicide utilized worked to individualize, pathologise and de-politicize suicide, dislocating the acts from the emotional worlds in which they occur (Marzetti et al., 2022). The narrow “momentary” focus of these policies centered “death prevention at the expense of considering ways of making individual lives more liveable” (Marzetti et al., 2022). We argue that in a similar manner, recent UK NHS interventions have focused on trying to improve the resilience of, or “fix,” individual nurses (e.g., through individualized interventions made under the national offer during COVID-19) rather than focus improvements on environmental factors and systems, such as workforce supply issues within the NHS, or trying to move away from problems generated by efficiency focused models of nursing where nursing work is viewed as a series of tasks to be completed (Leary, 2019). The adoption of individual-based resilience frameworks runs contrary to many perspectives, such as those within behavioral science, which have provided substantive evidence of the ecological causes of human behavior, with even personality traits resulting from environmental causes such as positive reinforcement of certain responses to distressing stimuli (Biglan & Hayes, 2015; Chiesa, 1992; Gore et al., 2013; Skinner, 1971). Holistic approaches, as opposed to psychocentric, can be viewed as having far greater benefits as they tend to include environmental, cultural, political, historical and biographical contexts enabling longer perspectives and solutions (Marsh, 2010).

Traynor (2017, p. 12) critiques nursing resilience studies and interventions for operating under a “tacit acknowledgement” that “the workplace experienced by nurses is so dysfunctional that it is better to invest energy in devising personal approaches to coping than investigating or challenging the causes of dysfunction.” Traynor (2017) goes on to suggest most resilience-based interventions and research within the NHS help to support the status quo through avoiding crises that might work to effect change in policy and thus argues that nursing-based resilience research has silenced any potential for agency or activism (Traynor, 2018). As our data have shown, nurses who are experiencing high levels of work-related stress, due to a lack of resources or ethical and emotional challenges during COVID-19 working conditions, “reported feeling that it is their ‘fault’ because they have not implemented ‘resilience training’ adequately or been ‘resilient enough’ with the consequence that resilience can be used as ‘another stick to beat [staff] with’ (Maben & Bridges, 2020). The dangers inherent in this construction of resilience can be viewed as originating in the notion of self-actualization, or becoming ones” ideal version of the self. This inherently involves understanding oneself as dis-preferred in some way and needing to be “fixed” which excludes the idea that “normal” psychological functioning is flawed as many people contend with a spectrum of illnesses (Riley et al., 2019). The acquisition of resilience can thus be viewed as a never-ending project, one can always become “better” and more resilient. The emphasis is truly placed on the individual to question “what’s the matter with me?.” Gill and Orgad (2019) found that those without the required resources (economic, physical and psychological) to access resilience, and who displayed a lack of resilience, became disposable. Recently, Gill and Orgad (2022) have noted that notions of self-confidence have been presented specifically to women, as the solution to a wide range of issues across many spheres of life, from the welfare system to consumer culture, body image, the workplace, parenting, education and sex and relationship advice. Rather than identifying the root causes of structural inequality, confidence culture reframes social injustices in terms of internal obstacles and personal deficits through, for example, familiar phrases such as “We do this to ourselves.” Gill and Orgad (2022) have argued that “we don’t need more emphasis on blaming and changing women, we need to change the world.”

## Limitations and Strengths

We acknowledge the small size of our sample of three case studies which were drawn from our larger data set of participants (*n* = 25 at two time points) in the ICON qualitative study. The nurses’ working locations were restricted to England, Wales and Scotland, which should be considered when interpreting the findings. Although this may be considered a strength as comparatively, the UK did experience high levels of COVID-19 infection and death rates. Our purposive sampling did enable us to gain a wide range of experiences from a variety of nurses who differed by attributes such as age, and speciality. However, our sample only contained one man and three nurses of ethnic minority background. By presenting the three case studies as exemplars, (reflecting the adversity and stressors nurses encountered in their COVID-19 working conditions as well as perceptions of their own resilience) this article has analyzed how the concept of resilience has affected three nurses’ emotional and mental wellbeing and care delivery during COVID-19. Our use of case studies to test, explore and refine theoretical ideas has given our analysis depth, gestalt and robustness (Hollway & Jefferson, 2013).

## Conclusion

The COVID-19 pandemic brought highly altered working environments for the majority of nurses, which left many vulnerable to poor mental health outcomes with feeling they were “not resilient enough.” We urge caution regarding the continuing use of the term resilience in the health context because of the wider meaning it has assumed in contemporary, neo-liberal, consumer-based society and its positioning within wider self-help and self-actualization discourses. Workplace systems need to be modified or created to support nurses to eliminate any prospect of labeling workplace distress being due only to individual failings or low levels of resilience. Within nursing, organizational problems were exacerbated by the COVID-19 pandemic and we argue that employing organizations also need to be made accountable for systemic failings that contribute to the demise of an individual’s sense of resilience. Optimally we would argue that better pandemic preparation needs to occur within health services, but also within governments. We call for more work to be done at a national level within the UK to address the expectations for nurses to be stoic and resilient in order to cope with their work, rather than an organizational responsibility to organize and plan work compassionately (Maben & Conolly, 2022). Systemic change is needed to support, create and foster nurses’ well-being within health services at all times but particularly in the extreme working environments of a global pandemic.

## Notes

1. In the UK, nurses directly employed by the NHS have standardized grading, ranging from band two (e.g., healthcare support worker) to band nine (senior manager). Band five is the entry point for most registered healthcare professionals, including nurses. For those not directly employed by the NHS, we asked them to record their NHS-equivalent banding.

## References

[bibr1-23333936221094862] Allen J. Diefendorff J. Ma Y. (2014). Differences in emotional labor across cultures: A comparison of Chinese and U.S. service workers. Journal of Business and Psychology, 29, 21–35. 10.1007/s10869-013-9288-7

[bibr2-23333936221094862] Anderson K. M. Renner L. M. Danis F. S. (2012). Recovery: Resilience and growth in the aftermath of domestic violence. Violence Against Women, 18(11), 1279–1299. 10.1177/1077801212470543 23334815

[bibr3-23333936221094862] Biglan A. Hayes S. C. (2015). Functional contextualism and contextual behavioral science. In Zettle R. Hayes S. C. Barnes-Holmes D. Biglan T. (Eds.), Wiley handbook of contextual behavioral science (pp. 161–175). Wiley-Blackwell.

[bibr4-23333936221094862] British Academy. (2021). The COVID decade: Understanding the long-term societal impacts of COVID-19. The British Academy.

[bibr5-23333936221094862] Charmaz K. (2006). Constructing grounded theory. Sage.

[bibr6-23333936221094862] Chiesa M. (1992). Radical behaviorism and scientific frameworks: From mechanistic to relational accounts. American Psychologist, 47(11), 1287–1299. 10.1037/0003-066x.47.11.1287 1482000

[bibr7-23333936221094862] Christodoulou-Fella M. Middleton N. Papathanassoglou E. D. E. Karanikola M. N. K. (2017). Exploration of the association between nurses’ moral distress and secondary traumatic stress syndrome: Implications for patient safety in mental health services. BioMed Research International, 2017, 1908712–1908719. 10.1155/2017/1908712 29209622 PMC5676344

[bibr8-23333936221094862] Cooper A. L. Brown J. A. Rees C. S. Leslie G. D. (2020). Nurse resilience: A concept analysis. International Journal of Mental Health Nursing, 29, 553–575. 10.1111/inm.12721 32227411

[bibr9-23333936221094862] Couper K. Murrells T. Sanders J. Anderson J. E. Blake H. Kelly D. Kent B. Maben J. Rafferty A. M. Taylor R. M. Harris R. (2022). The impact of COVID-19 on the wellbeing of the UK nursing and midwifery workforce during the first pandemic wave: A longitudinal survey study. International Journal of Nursing Studies, 127, 1–14. 10.1016/j.ijnurstu.2021.104155 PMC8673915 35093740

[bibr10-23333936221094862] Delgado C. Upton D. Ranse K. Furness T. Foster K. (2017). Nurses’ resilience and the emotional labour of nursing work: An integrative review of empirical literature. International Journal of Nursing Studies, 70, 71–88. 10.1016/j.ijnurstu.2017.02.008 28235694

[bibr11-23333936221094862] Douglas J. D. (1967). The social meaning of suicide. Princeton University Press.

[bibr12-23333936221094862] Edvardson D. Rasmussen B. Riessman C. (2003). Ward atmospheres of horror and healing: A comparative analysis of narrative. Health, 7(4), 377–396. 10.1177/13634593030074001

[bibr13-23333936221094862] Elliot V. (2018). Thinking about the coding process in qualitative data analysis. The Qualitative Report, 23(11), 2850–2861. https://nsuworks.nova.edu/tqr/vol23/iss11/14

[bibr14-23333936221094862] Flyvbjerg B. (2006). Five misunderstandings about case-Study Research. Qualitative Inquiry, 12(2), 219–245. 10.1177/1077800405284363

[bibr15-23333936221094862] Foster K. Roche M. Delgado C. Cuzzillo C. Giandinoto J. A. Furness T. (2019). Resilience and mental health nursing: An integrative review of international literature. International Journal of Mental Health Nursing, 28(1), 71–85. 10.1111/inm.12548 30294937

[bibr16-23333936221094862] Foureur M. Besley K. Burton G. Yu N. Crisp J. (2013). Enhancing the resilience of nurses and midwives: Pilot of a mindfulnessbased program for increased health, sense of coherence and decreased depression, anxiety and stress. Contemporary Nurse, 45(1), 114–125. 10.5172/conu.2013.45.1.114 24099232

[bibr17-23333936221094862] Garmezy N. Masten A. S. Tellegen A. (1984). The study of stress and competence in children: A Building Block for Developmental Psychopathology. Child Development, 55, 97–111. 10.2307/1129837 6705637

[bibr18-23333936221094862] Gill R. Orgad S. (2019). The amazing bounce-backable woman: Resilience and the psychological turn in neoliberalism. Sociological Research Online, 23(2), 477–495. 10.1177/1360780418769673

[bibr19-23333936221094862] Gill R. Orgad S. (2022). Confidence culture tells women to be more self-assured – but ignores the real problems. The Conversation. https://theconversation.com/confidence-culture-tells-women-to-be-more-self-assured-but-ignores-the-real-problems-176864

[bibr20-23333936221094862] Gore N. McGill P. Toogood S. Allen D. Hughes C. Baker P. Hastings P. Noone S. Denne D. (2013). Definition and scope for positive behavioural support. International Journal of Positive Behavioural Support, 3(2), 14–23.

[bibr21-23333936221094862] Gorse C. Johnston D. Pritchard M. (2012). A Dictionary of construction, surveying, and civil engineering. Oxford University Press.

[bibr22-23333936221094862] Greenberg N. Weston D. Hall C. Caulfield T. Williamson V. Fong K. (2021). Mental health of staff working in intensive care during COVID-19. Occupational Medicine, 71, 62–67. 10.1093/occmed/kqaa220 33434920 PMC7928568

[bibr23-23333936221094862] Greenhalgh T. Russell J. Swinglehurst D. (2005). Narrative methods in quality improvement research. BMJ Quality and Safety, 14(6). 10.1136/qshc.2005.014712 PMC1744090 16326792

[bibr24-23333936221094862] Guba E. G. Lincoln Y. S. (1994). Competing paradigms in qualitative research. In Denzin N. K. Lincoln Y. S. (Eds.), Handbook of qualitative research (pp. 105–117). SAGE.

[bibr25-23333936221094862] Guest R. Craig A. Nicolson Perry K. (2015). Resilience following spinal cord injury. Rehabilitation Psychology, 60(4), 311–321. 10.1038/sc.2013.159 26348699

[bibr26-23333936221094862] Happell B. Dwyer T. Reid-Searl K. Burke K. J. Caperchione C. M. Gaskin C. J. (2013). Nurses and stress: Recognizing causes and seeking solutions. Journal of Nursing Management, 21(4), 638–647. 10.1111/jonm.12037 23700980

[bibr27-23333936221094862] Harding J. (2006). Questioning the subject in biographical interviewing. Sociological Research Online, 11(3). http://www.socresonline.org.uk/11/3/harding.html

[bibr28-23333936221094862] Health and Social Care Select Committee. (2021). Workforce burnout and resilience in the NHS and social care. https://committees.parliament.uk/work/494/workforce-burnout-and-resilience-in-the-nhs-and-social-care/

[bibr29-23333936221094862] Hollway W. Jefferson T. (2013). Doing qualitative research differently: Free association, narrative and the interview method (2nd ed.). SAGE.

[bibr30-23333936221094862] Hsieh H. F. Hung Y. T. Wang H. H. Ma S. C. Chang S. C. (2016). Factors of resilience in emergency department nurses who have experienced workplace violence in Taiwan. Journal of Nursing Scholarship, 48(1), 23–30. 10.1111/jnu.12177 26580490

[bibr31-23333936221094862] Jackson J. Anderson J. E. Maben J. (2021). What is nursing work? A meta-narrative review and integrated framework. International Journal of Nursing Studies, 122(2), 103944. 10.1016/j.ijnurstu.2021.103944 34325358

[bibr32-23333936221094862] Jones A. Kelly D. (2014). Deafening silence? Time to reconsider whether organisations are silent or deaf when things go wrong. BMJ Quality & Safety, 23, 709–713. https://doi.org.10.1136/bmjqs-2013-002718 10.1136/bmjqs-2013-002718 25015116

[bibr33-23333936221094862] Kinman G. Teoh K. Harriss A. (2020). The mental health and wellbeing of nurses and midwives in the United Kingdom (Technical Report). Society of Occupational Medicine.

[bibr34-23333936221094862] Kinman G. Leggetter S. (2016). Emotional labour and wellbeing: What protects nurses? Healthcare, 4(4), 89–12. 10.3390/healthcare4040089 PMC5198131 27916880

[bibr35-23333936221094862] Kirk K. Cohen L. Edgley A. Timmons S. (2021). “I don’t have any emotions”: An ethnography of emotional labour and feeling rules in the emergency department. Journal of Advanced Nursing, 77, 1956–1967. 10.1111/jan.14765 33576110

[bibr36-23333936221094862] Kunzler A. M. Helmreich I. Chmitorz A. König J. Binder H. Wessa M. Lieb K. (2020). Psychological interventions to foster resilience in healthcare professionals (Review). Cochrane Database of Systematic Reviews, 7, CD012527. 10.1002/14651858.CD012527.pub2 PMC8121081 32627860

[bibr37-23333936221094862] Kvale S. (1999). The psychoanalytic interview as qualitative research. Qualitative Inquiry, 5, 87–113. 10.1177/107780049900500105

[bibr38-23333936221094862] Labrague L. J. de Los Santos J. A. A. (2020). COVID-19 anxiety among front-line nurses: Predictive role of organisational support, personal resilience and social support. Journal of Nurse Management, 28, 1653–1661. 10.1111/jonm.13121 PMC7436313 32770780

[bibr39-23333936221094862] Lapum J. Nguyen M. Fredericks S. Lai S. McShane J. (2021). “Goodbye . . . through a glass door”: Emotional experiences of working in COVID-19 Acute Care Hospital Environments. Canadian Journal of Nursing Research, 53(1), 5–15. 10.1177/0844562120982420 PMC7754157 33342299

[bibr40-23333936221094862] Leary A. (2019). The healthcare workforce should be shaped by outcomes, rather than outputs. BMJ Opinion. https://blogs.bmj.com/bmj/2019/05/31/alison-leary-the-healthcare-workforce-should-be-shaped-by-outcomes-rather-than-outputs/

[bibr41-23333936221094862] Maben J. Bridges J. (2020). Covid-19: Supporting nurses’ psychological and mental health. Journal of Clinical Nursing, 29(15–16), 2742–2750. 10.1111/jocn.15307 32320509 PMC7264545

[bibr42-23333936221094862] Maben J. Conolly A. (in press). Lessons for structure, workplace planning and responding to emergencies from nurses in the COVID-19 pandemic. In Williams R. Kemp V. Porter K. Healing T. Drury J. (Eds.), Pandemics, major incidents and Mental Health: The Psychosocial and Mental Health Aspects of Health Emergencies. Cambridge University Press.

[bibr43-23333936221094862] Maben J. Conolly A. Abrams R. Rowland E. Harris R. Kelly D. Kent B. Couper K. (2022). ‘You can’t walk through water without getting wet’: Exploring nurse distress and psychological health needs during COVID-19: A longitudinal qualitative study. International Journal of Nursing Studies. Advance online publication. 10.1016/j.ijnurstu.2022.104242 PMC8976696 35525086

[bibr44-23333936221094862] Maben J. Latter S. Clark J. (2006). The theory-practice gap: Impact of professional-bureaucratic work conflict on newly-qualified nurses. Journal of Advanced Nursing, 55(4), 465–77. 10.1111/j.1365-2648.2006.03939.x 16866842

[bibr45-23333936221094862] Marsh I. (2010). Suicide: Foucault, history and truth. Cambridge University Press.

[bibr46-23333936221094862] Marzetti H. Oaten A. Chandler A. (2022). Self-inflicted. Deliberate. Death-intentioned. A critical policy analysis of UK suicide prevention policies 2009-2019. Journal of Public Mental Health, 21(1), 4–14. https//doi.org/10.1108/JPMH-09-2021-0113

[bibr47-23333936221094862] Maslow A. H. (1943). A theory of human motivation. Psychological Review, 50(4), 370–396.

[bibr48-23333936221094862] McDonald G. Jackson D. Wilkes L. Vickers M. (2013). Personal resilience in nurses and midwives: Effects of a work-based educational intervention. Contemporary Nursing, 45(1), 134–143. 10.5172/conu.2013.45.1.134 24099234

[bibr49-23333936221094862] Mealer M. Jones J. Meek P. (2017). Factors affecting resilience and development of posttraumatic stress disorder in critical care nurses. American Journal of Critical Care, 26(3), 184–192. 10.4037/ajcc2017798 28461539 PMC5685839

[bibr50-23333936221094862] Mills J. Bonner A. Francis K. (2006). The development of constructivist grounded theory. International Journal of Qualitative Methods, 5(1), 25–35. 10.1177/160940690600500103

[bibr51-23333936221094862] Mitchell J. C. (2000). Case and situation analysis. In Gomm R. Hammersley P. Foster P. (Eds.), Case study method (pp. 165–186). SAGE.

[bibr52-23333936221094862] Morse J. Kent-Marvick J. Barry L. Harvey J. Narkie Okang E. Rudd E. Wang C. Williams M. (2021). Developing the resilience framework for nursing and healthcare. Global Qualitative Nursing Research, 8, 1–21. 10.1177/23333936211005475 PMC8020405 33869667

[bibr53-23333936221094862] Ohta R. Matsuzaki Y. Itamochi S. (2020). Overcoming the challenge of COVID-19: A grounded theory approach to rural nurses’ experiences. Journal of General Family Medicine, 00, 1–7. 10.1002/jgf2.410 PMC7753682 33362984

[bibr54-23333936221094862] Riessman C. K. (1993). Narrative analysis. Sage.

[bibr55-23333936221094862] Riessman C. K. (2002). Analysis of personal narratives. In Gubrium J. Holstein J. (Eds.), Handbook of interview research (pp. 695–710). Sage.

[bibr56-23333936221094862] Riley S. Evans A. Anderson E. Robson M. (2019). The gendered nature of self-help. Feminism & Psychology, 29(1), 3–18. 10.1177/0959353519826162

[bibr57-23333936221094862] Rimke H. (2016). Introduction – Mental and emotional distress as a social justice issue: Beyond psychocentrism. Studies in Social Justice, 10(1), 4–17. 10.26522/ssj.v10i1.1407

[bibr58-23333936221094862] Robinson O. C. (2014). Sampling in interview-based qualitative research: A theoretical and practical guide. Qualitative Research in Psychology, 11(1), 25–41. 10.1080/14780887.2013.801543

[bibr59-23333936221094862] Skinner B. F. (1971). Beyond freedom and dignity. Knopf.

[bibr60-23333936221094862] Taylor R. A. (2019). Contemporary issues: Resilience training alone is an incomplete intervention. Nurse Education Today, 78, 10–13. 31029952 10.1016/j.nedt.2019.03.014

[bibr61-23333936221094862] Thomson R. Bell R. Holland J. Henderson S. McGrellis S. Sharpe S. (2002). Critical moments: Choice, chance and opportunity in young people’s narratives of transition. Sociology, 36(2), 335–354. 10.1177/0038038502036002006

[bibr62-23333936221094862] Traynor M. (2017). Critical resilience for nurses. Routledge.

[bibr63-23333936221094862] Traynor M. (2018). Guest editorial: What’s wrong with resilience. Journal of Research in Nursing, 23(1), 5–8. 10.1177/1744987117751458 34394401 PMC7932251

[bibr64-23333936221094862] Ustun G. (2021). COVID-19 pandemic and mental health of nurses. In Firstenberg M. Stawicki S. Papadimos T. (Eds.), Contemporary developments and perspectives in international health security volume 2 (pp. 167–184). 10.5772/intechopen.96084IntechOpen.

[bibr65-23333936221094862] Virkstis K. Herleth A. Langr M. (2018). Cracks in the foundation of the care environment undermine nurse resilience. The Journal of Nursing Administration, 48(12), 597–599. 10.1097/nna.0000000000000687 30431511

[bibr66-23333936221094862] Walker L. Avant K. (2011). Strategies for theory construction in nursing (5th ed.). Prentice Hall.

[bibr67-23333936221094862] World Health Organization (WHO). (2006). The World Health Report—Working Together for Health. World Health Organization, Geneva.

[bibr68-23333936221094862] World Health Organization (WHO). (2021). Nursing and midwifery: Key facts. https://www.who.int/news-room/fact-sheets/detail/nursing-and-midwifery

[bibr69-23333936221094862] Zizek S. (2005). The metastases of enjoyment: On women and causality (2nd ed.). Verso Books.

